# Epilepsy in Pediatric Patients—Evaluation of Brain Structures’ Volume Using VolBrain Software

**DOI:** 10.3390/jcm11164657

**Published:** 2022-08-09

**Authors:** Magdalena Maria Woźniak, Monika Zbroja, Małgorzata Matuszek, Olga Pustelniak, Weronika Cyranka, Katarzyna Drelich, Ewa Kopyto, Andrzej Materniak, Tomasz Słomka, Maciej Cebula, Agnieszka Brodzisz

**Affiliations:** 1 Department of Pediatric Radiology, Medical University of Lublin, 20-093 Lublin, Poland; 2 Students’ Scientific Society at the Department of Pediatric Radiology, Medical University of Lublin, 20-093 Lublin, Poland; 3 Department of Medical Informatics and Statistics with E-Learning Lab, Medical University of Lublin, 20-090 Lublin, Poland; 4 Department of Radiology and Nuclear Medicine, Medical University of Silesia, 40-752 Katowice, Poland

**Keywords:** MR imaging, volBrain software, epilepsy, volumetric analysis

## Abstract

Epilepsy is one of the most frequent serious brain disorders. Approximately 30,000 of the 150,000 children and adolescents who experience unprovoked seizures are diagnosed with epilepsy each year. Magnetic resonance imaging is the method of choice in diagnosing and monitoring patients with this condition. However, one very effective tool using MR images is volBrain software, which automatically generates information about the volume of brain structures. A total of 57 consecutive patients (study group) suffering from epilepsy and 34 healthy patients (control group) who underwent MR examination qualified for the study. Images were then evaluated by volBrain. Results showed atrophy of the brain and particular structures—GM, cerebrum, cerebellum, brainstem, putamen, thalamus, hippocampus and nucleus accumbens volume. Moreover, the statistically significant difference in the volume between the study and the control group was found for brain, lateral ventricle and putamen. A volumetric analysis of the CNS in children with epilepsy confirms a decrease in the volume of brain tissue. A volumetric assessment of brain structures based on MR data has the potential to be a useful diagnostic tool in children with epilepsy and can be implemented in clinical work; however, further studies are necessary to enhance the effectiveness of this software.

## 1. Introduction

Epilepsy is one of the most frequent serious brain disorders. It is a combination of somatic, vegetative, and psychiatric symptoms resulting from both morphological and metabolic changes in the brain. It is characterized by a persistent predisposition to generate seizures. The prevalence of epilepsy among the pediatric population is the highest during the first year of life [1]. Approximately 30,000 of the 150,000 children and adolescents who experience unprovoked seizures are diagnosed with epilepsy each year [2]. Epilepsy that occurs in childhood is a heterogeneous disorder with different etiology, clinical manifestation, severity and prognosis [3]. The differentiation of epilepsy is, with a range of clinical conditions, characterized by transient changes in consciousness and/or behavior [1].

Children show cognitive impairment, possibly due to changes in the brain structures following seizures or epileptic dysfunction. Discrete changes in the brain architecture, function or biochemistry can be assessed by imaging studies, which detect focal changes in 21–37% of patients with epileptic seizures [4,5].

Magnetic resonance (MR) is the method of choice for diagnosing epilepsy. The detection of structural changes in the central nervous system (CNS) in this examination, correlating with other diagnostic findings, significantly increases the chance of successful surgical treatment of epilepsy and could possibly help to define or to clarify the etiology of epileptic seizures. MR imaging performed in accordance with a standard protocol enables the detection of focal lesions (e.g., tumors, vascular malformations, cortical dysplasia), while the use of appropriate MR protocols (Table 1) significantly multiplies the examination’s sensitivity in terms of finding foci of cortical dysplasia and hippocampal sclerosis [5,6,7]. The application of a specific protocol for suspected epilepsy increases the success rate of confirmation of lesions in the patients studied from 49% to 72% [4].

However, chronic epilepsy may be associated with the occurrence of structural and volumetric changes in the brain both within and beyond the epileptogenic zone. Cross-sectional MR imaging studies in patients with intractable epilepsy have reported significant cerebellar and cerebral volume reduction as well as atrophy of the hippocampus or brain cortex in children and adults [10,11]. Atrophy in epileptic patients is a consequence of disease-related factors such as hypoxia and treatment with antiepileptic drugs [12]. There are several studies that have reported atrophy of brain structures in patients with epilepsy based on posthumous, pneumoencephalography, computed tomography and MR imaging [13]. Up to now, manual segmentation has been the method of choice for the accurate analysis of specific brain structures. Nevertheless, this task has proven to be time-consuming, leading to its limited use in clinical practice. The increasing daily volume of neuroimaging data has prompted the search of automated and objective algorithms, such as volBrain. The volBrain software solution is a free online system for brain MR volumetry. It is designed to help researchers automatically obtain information about the volume of brain structures from MR data, without the necessity of having any of the infrastructure in their local centers [14].

By following this thought, it was our aim to evaluate the volume of selected brain structures in the pediatric population and assess whether differences exist between individuals with epilepsy and healthy volunteers in terms of brain atrophy.

Four hypotheses were formulated:

**Hypothesis** **1** **(H1)**. *During epilepsy, there is an atrophy of brain tissue that reflects in the decrease in volume of particular brain structures*.

**Hypothesis** **2** **(H2)**. *There is a significant difference in particular structures’ volumes between the study and control group*.

**Hypothesis** **3** **(H3)**. *There is a relationship between the volume of individual CNS structures and the presence of comorbidities in a group of children with epilepsy*.

**Hypothesis** **4** **(H4)**. *VolBrain software is a tool for parameterization of brain lesions in the course of epilepsy*.

## 2. Materials and Methods

**Figure 1:**
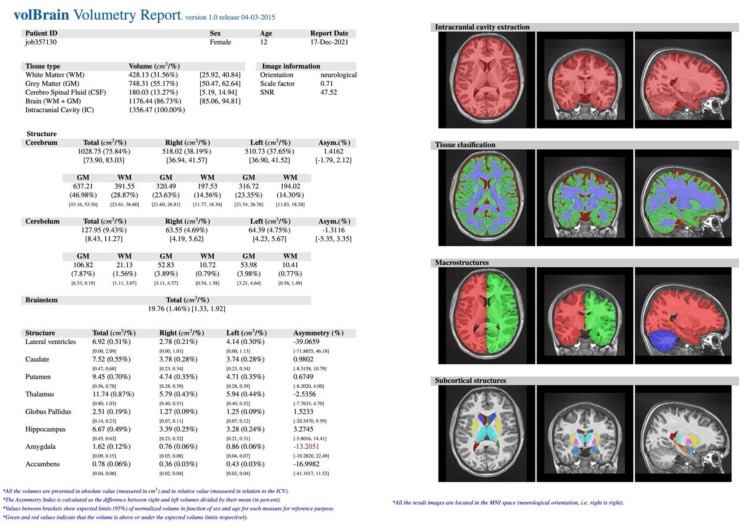
Example of volBrain pdf report. The report consisted of several parts. At the top, there is hidden patient data with a randomly assigned number plus age and gender. The next section lists the measured structures: (**A**) macrostructures such as WM, GM, CSF, IC, cerebrum, cerebellum, brainstem, and subcortical, (**B**) microstructures such as putamen, caudate, pallidum, thalamus, hippocampus, amygdala, and nucleus accumbens. Values are expressed in cubic centimeters and percentages. On the right, there is a visual example of the segmentation process (axial, sagittal, and coronal views) to certify its quality. Source: own study.

In our retrospective study, the study (S) consisted of 57 consecutive patients (F = 30, M = 27) suffering from epilepsy, regardless of its type and disease duration, who underwent head MR examination without contrast agent administration between 2018 and 2021 at the Children’s Hospital of the Medical University of Lublin in Poland. Their mean age was 8.94 ± 4.72 years (min. 4 months, max. 17 years). The exclusion criteria included the most common causes of secondary epilepsies such as post-traumatic, hemorrhagic, ischemic-hypoxic lesions and brain structure deformations such as hydrocephalus, tumors, arteriovenous malformation or developmental disorders. The control (C) group included 34 healthy patients (F = 15, M = 19) without epilepsy with mean age 9.24 ± 5.44 years (min. 6 months, max. 17 years) who underwent head MR examination for reasons other than epilepsy. All examinations were performed using Siemens Aera 48-channel 1.5T MRI scanner according to a dedicated protocol (epilepsy-specific protocol) (T1WI isotropic 3D-sequence, FLAIR, T2WI in axial, coronal-perpendicular to the long axis of the hippocampus, and transverse sequence). The study was based on assessment with the use of T1-weighted ISO 3D MRI images (*n* = 91). The process of obtaining the required data consisted of several steps. Initially, images of certain patients were exported from the local Picture Archiving and Communication System (PACS). Then, the scans in DICOM format (.dcm) were converted into NIFTI format (.nii), using free MRIcron software. Further steps involved creating a free account and registering on the volBrain website (www.volbrain.upv.es/ accessed on 1 May 2022). Then, individual scans in NIFTI format were uploaded to the volBrain. The software rendered an automatic, quantitative analysis of the MR data. Processing time for a single image averaged 14 min (depending on how occupied the servers were at the particular time). The volBrain pipeline is designed to enhance the quality of the input images and set them in a geometric space and intensity to segment and distinguish the various structures and tissues. The segmentation process consists of the following steps: spatially adaptive non-local means denoising, rough inhomogeneity correction, affine registration to MNI space, fine SPM-based inhomogeneity correction, intensity normalization, non-local Intracranial Cavity Extraction (NICE), tissue classification, non-local hemisphere segmentation (NABS) and non-local subcortical structure segmentation, respectively [15]. Once the process was finished, automatically generated segmentation PDF reports (Figure 1) were provided via email. The reports contain objectively measured tissue volumes such as white matter (WM), grey matter (GM), CSF (cerebrospinal fluid), intracranial cavity (IC), cerebrum, cerebellum, brainstem, lateral ventricles, and subcortical structures such as putamen, caudate, pallidum, thalamus, hippocampus, amygdala, and nucleus accumbens. Tissue volumes are presented as absolute values measured in cubic centimeters (cm^3^) and as the ratio of the given structure’s volume to the IC volume (that covers 100%) expressed in percentage. An additional parameter is the asymmetry index, which is the difference between the volume of the right and left structures divided by their mean volume, expressed in percentage. Reports were subjected to substantive assessment. Statistical analysis was performed using percentage values sourced from the volBrain program. The normality test for the quantitative variables was performed by the Shapiro–Wilk W test. For all the statistical analyses, when *p* < 0.05, the test result was considered statistically significant. Then the null hypothesis was rejected. If the normality of the distribution was not rejected, the parametric test statistic for the two independent groups (Student’s *t*-test) was implemented. Otherwise, the Mann–Whitney U test was used. All the statistical analyses were conducted with TIBCO Software Inc. (2020). Data Science Workbench, version 14. [http://tibco.com accessed on 1 May 2022]. The final phase was based on a comparative analysis of the structures’ volume differences between the S group and the C group.

## 3. Results

**Figure 2:**
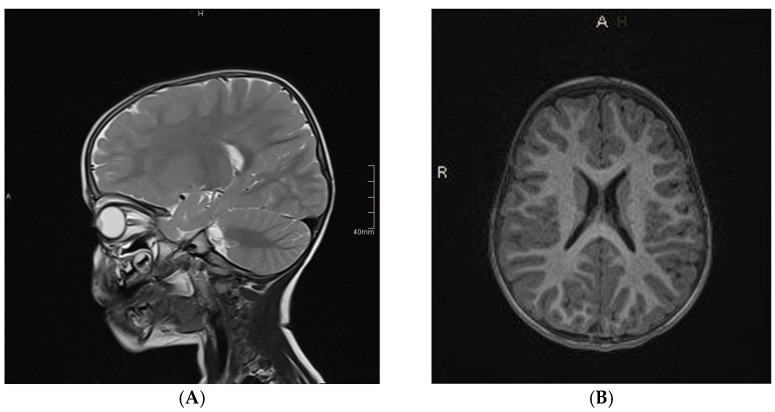
Head MR imaging (T2−weighted (**A**) and T1−weighted (**B**) images) of 2.5−year−old boy with a lack of eye contact and abnormal eye movements. The MR examination was normal. Source: own study.

**Figure 3:**
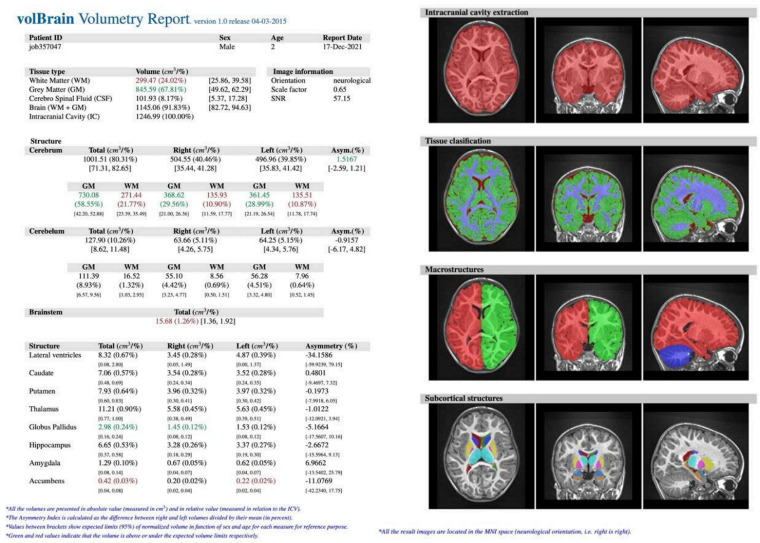
VolBrain analysis of head MR of a 2.5−year−old boy—no relevant abnormalities were detected. Source: own study.

In 34 patients (C group), there were no relevant lesions in the brain in the MR examination (example in Figure 2), although nearly half of them presented neurological symptoms such as dizziness, abnormal eye movements, or numbness. Moreover, the volBrain software did not confirm any abnormal volumes of brain tissue or particular structures (example in Figure 3).

**Figure 4:**
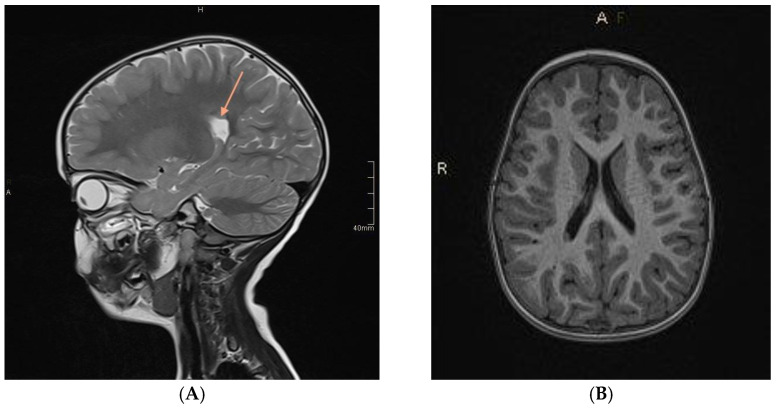
Head MR imaging (T2−weighted (**A**) and T1−weighted (**B**) images) of a 3−year−old boy after the third episode of seizures. MR examination showed a single focus of heterotopia. Source: own study.

A total of 57 patients had symptoms indicative of epilepsy seizures. Characteristic structural changes such as heterotrophy or atrophy of the hippocampus were revealed on MR in all of them. An example of an MR examination is shown on Figure 4.

**Figure 5:**
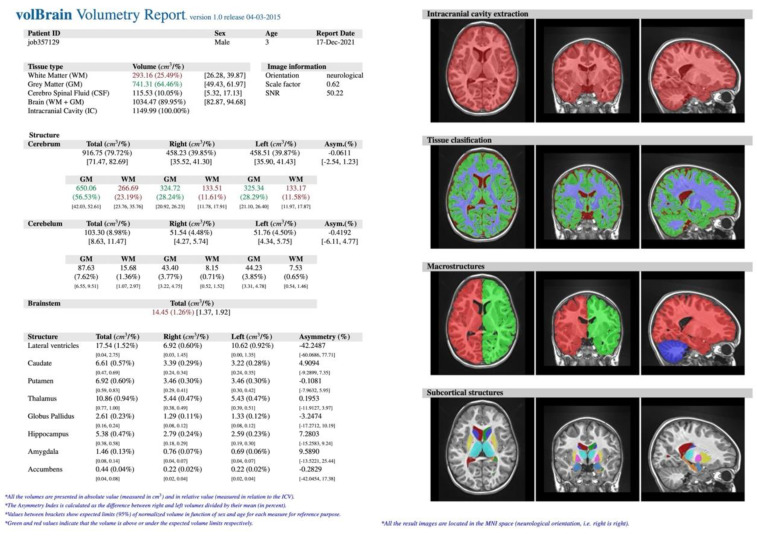
VolBrain analysis of head MR of a 3−year−old boy. Source: own study.

Images were then evaluated with volBrain software, which quantitatively confirmed former MR findings. In the example (Figure 5), there is brain atrophy with a lower value of GM, cerebrum, cerebellum, brainstem, putamen, globus pallidus, hippocampus, nucleus accumbens, and amygdala volume, whereas volumes of WM, CSF, and thalamus increased in comparison to the previous case (Figure 2 and Figure 3).

### Statistical Analysis

The statistical analysis was then conducted on the basis of volBrain results, and the normality test for the quantitative variables was performed by the Shapiro–Wilk W test (Table 2).

However, due to the rejection of the normality of the distribution of the variables (in C or S groups) the non-parametric Mann–Whitney U test was applied to test the difference between the two groups (Table 3).

The statistically significant differences between the S and the C group were found for:

1. Brain

**Figure 6:**
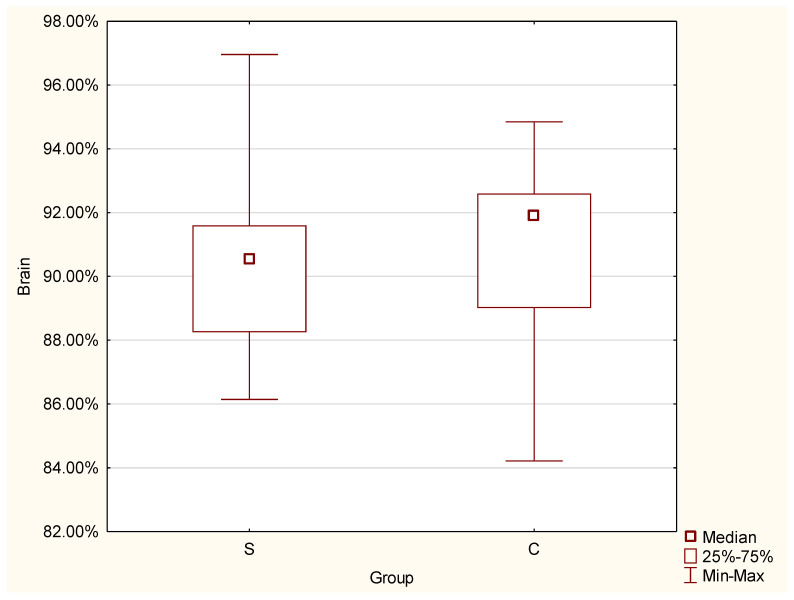
Differences in brain volume between the control and study group.

For brain, the test indicated a significant difference in the volume. A greater result was found for the C group (Mdn = 91.02%) than for the S group (Mdn = 90.53%), (Z = −2.19, *p* = 0.029 *) (Figure 6).

2. Lateral ventricles

**Figure 7:**
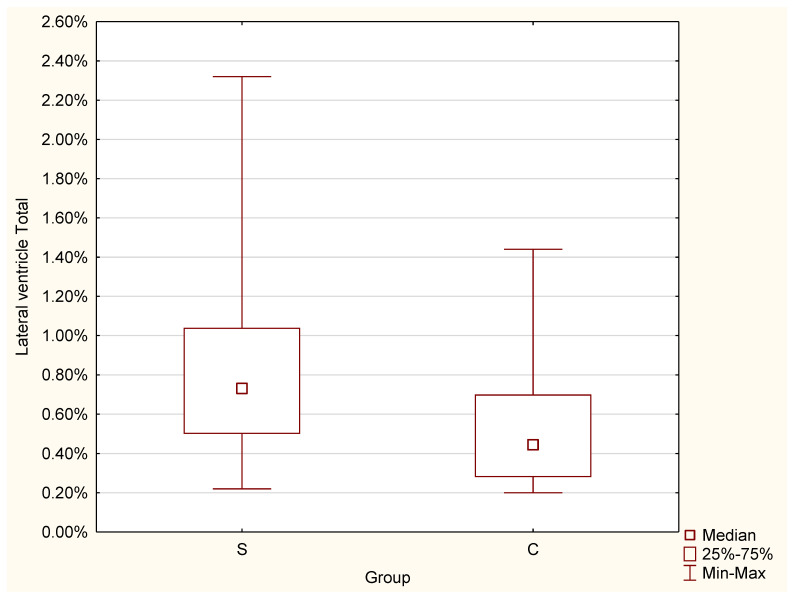
Differences in lateral ventricles volume between the C and S groups.

The significant difference was proven by the conducted test for lateral ventricles, and a greater result was found for the S group (Mdn = 0.73%) than for the control group (Mdn = 0.55%), (Z = 3.25, *p* = 0.001 *) (Figure 7).

3. Putamen

**Figure 8:**
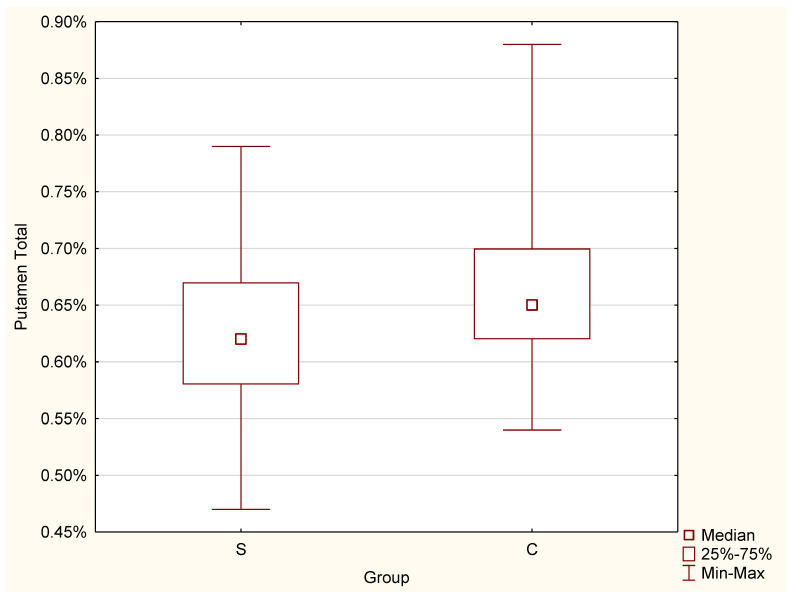
Differences in putamen volume between the C and S groups.

The conducted test for putamen indicated that the value was greater for the control group (Mdn = 0.67%) than for the studied group (Mdn = 0.62%) (Z = −2.01, *p* = 0.044 *) (Figure 8).

The results for the other tested factors indicated non-significant differences between the groups (S and C). Nevertheless, there is a definite declining trend in the volume of remaining structures in patients with epilepsy as compared to healthy patients.

Next, the results from the volBrain program were analyzed according to the presence of comorbidities in a group of children with epilepsy. The following comorbidities/conditions were included in the analysis: prematurity, delayed development, cerebral palsy, the presence of arachnoid cyst, Recklinghausen’s disease, undefined CNS defects, mental disability, speech disorders, hearing loss and factor XII deficiency; however, due to the small number of distributed variables, the analysis was based on two variables—the absence or presence of comorbidities.

Although no significant differences were observed between the groups, patients with comorbidities showed bigger brain atrophy (90.11 vs. 90.55% of the IC), and greater volume of the lateral ventricles (0.98 vs. 0.69% of the IC) (Table 4).

In addition, EEG examinations of patients with epilepsy were also included in the analysis. Four groups were selected: normal EEG, paroxysmal lesions, generalized lesions and localized lesions. The results showed that there were no significant differences for all quantitative variables (volumes of individual structures) relative to normal EEG or EEG deviations. The study group was also divided according to the duration of the disease (group 1—up to 12 months, group 2—1–2 years, group 3—>2 years), which was then confronted with changes in volumes of the particular intracranial structures. Similarly, no significant relationships were found between disease duration and quantitative variables.

## 4. Discussion

The etiology of epileptic seizures is multifactorial: genetic, metabolic, and dependent on triggering immune factors [16]. Chromosomal abnormalities in which we observe epileptic seizures include 15q13.3 microdeletion syndrome, Angelman’s syndrome, Down’s syndrome, Klinefelter’s syndrome [17]. Seizures secondary to structural damage are also observed in many epilepsies. Central nervous system defects account for as much as 5% of epilepsy, most often starting early in life. Many of them present as a specific type of seizure. In lissencephaly and cortical dysplasia, we usually observe treatment-resistant flexion attacks. In the course of pachygyria, polymicrogyria, and also gray matter heterotopia, there are focal seizures. Agenesia and hypoplasia of the corpus callosum are the cause of flexion, focal and generalized seizures. The defects associated with epilepsy also include schizencephaly and Struge–Weber syndrome [18]. In our study, the most common changes were heterotopia and atrophy of the hippocampus.

Many studies support the hypothesis that the cause of epileptic seizures might be attributed to an imbalance between excitatory and inhibitory currents that include: gliosis, uncontrolled inflammation, an impaired blood–brain barrier (BBB), neurodegeneration, aberrant neurogenesis, axonal and dendritic plasticity, changes in neural circuits, structural and functional changes in receptors, ion channels, transporters and enzymes implicated in excitatory and inhibitory neurotransmission, reorganization of the extracellular matrix (ECM) and epigenetic reprogramming [19]. There is some evidence showing that impaired gamma-aminobutyric acid (GABA)-ergic inhibitory feedback influences the enhanced excitability, especially in the case of focal epilepsy [20].

In a single study from the Institute of Neurology in London, postmortem examinations revealed macroscopic abnormalities in 70% of epilepsy cases. These included: contusions, old infarcts, hippocampal sclerosis, cortical dysgenesis, vascular malformations, oligodendrogliomas, neurodegenerative brain diseases, and microcephaly [21]. The above-mentioned conditions are a cause of secondary epilepsy, but in addition, the study also confirmed reduced brain volumes in cases of sudden unexpected death due to epilepsy. Seizures may also be a consequence of acquired metabolic disorders, such as hypoglycemia, hyponatremia, renal failure or hypoparathyroidism [22], but our patients had no relevant information on such disorders. In one study, researchers identified and quantified 72 metabolites, 14 of which (especially monophosphate and O-acetylcholine) showed significant differences in concentrations between the patients with not-otherwise-specified epilepsy and healthy controls [23].

For several years, the effect of epilepsy on the volume of the brain structures has begun to be considered. To date, the volumetry of individual parts has been assessed based on post-mortem examinations, pneumoencephalography, computed tomography and magnetic resonance imaging. Studies in which traditional methods of measurement were used were analyzed [10,11,13].

The most common MR techniques to evaluate the abnormalities of the entire brain comprise the assessment of the anatomical structure (volumetric and morphometric MRI), white matter tissue properties (diffusion MRI), neuronal activation (functional MRI) and metabolite concentrations (MRSI) [24,25]. Functional changes caused by epilepsy have also become a research interest. fMRI provides neurophysiological and pathological evidence for the changes in epileptic patients’ brains. In general, functional MRI (fMRI) is used to detect changes in regional blood flow (brain perfusion) and metabolism that accompany regional brain activation [26]. Researchers have used resting-state fMRI to study the changes in resting spontaneous brain function in patients with temporal lobe epilepsy with cognitive impairment (TLE-CI) [27]. In one of the articles, we can read that major applications of fMRI in epilepsy include the localization of task-correlated language and memory function and also the localization of ictal and paroxysmal phenomena [26]. In the literature, we can also find investigations focused on epilepsy as a network disorder and in the context of comorbidities as well [24]. There are only a few reports on the use of MRI in detecting abnormalities within other brain structures, let alone relating to changes in their morphology or anatomy, including volume and density.

The study, carried out with MR imaging-based volume measurements, proves that multiple brain regions beyond the hippocampus are volumetrically affected in patients with mesial temporal lobe epilepsy with hippocampal sclerosis (MTLE + HS). Patients displayed significant volume reduction in the ipsilateral amygdala, thalamus, and cerebral WM. In addition, patients with left MTLE + HS displayed volume loss in the contralateral hippocampus and cerebellar GM bilaterally [28].

Another analysis of optimized voxel-based morphometry results showed a significantly smaller size of structures such as the rostrum and rostral body of corpus callosum and the left hippocampus compared to the C group. The left frontal lobe was significantly larger in the epilepsy group [29]. Our study showed the greater caudate and lateral ventricles volume. The results of the next study showed significant reductions in volume and GM concentration of the hippocampus [30]. In addition, it is assumed that the atrophy of the cerebellum is the most common symptom in patients suffering from epilepsy [31].

Based on our study, in which we used VolBrain software to analyze the volume of each CNS structure, we found that there is also a slight reduction in hippocampus and thalamus volume, but no difference in amygdala and globus pallidus volume in the S and C group.

There are a few studies in the literature that analyzed the volume of specific CNS structures in patients with epilepsy using VolBrain software, which is emphasized by the fact that it is an extremely innovative method. Contrary to our study, other authors included adult and pediatric patients in their analysis.

The first study involved an analysis that investigated the cerebellar substructure alterations and the association with clinical factors and cognitive scores among refractory unilateral TLE patients. The study included 48 patients aged 14 to 60 years who suffered from drug-resistant unilateral temporal epilepsy. The total volumes of crus I, crus II, and IX were significantly smaller on both sides; however, the GM volumes of cerebellar lobules showed a reduction in crus III and IX ipsilateral and crus II contralateral. The duration of epilepsy has no effect on the GM volumes of contralateral crus II. A decrease in the volume of the GM volumes of contralateral crus II resulted in significant deviations in the cognitive scores [13].

Subsequent studies were devoted to the analysis of the volume of the hippocampus and the subfield of the hippocampus in patients with TLE. Based on the analysis, it was found that the volume of the hippocampus decreased in the course of TLE. Moreover, hippocampal volumes ipsilateral to the hippocampal sclerosis side were significantly reduced compared with controls and also with the non-lesional side [32]. Additionally, some patients with left exhibited ipsilateral hippocampal atrophy and segmental volume depletion of the cornu ammonis, dentate gyrus, and stratum radiatum-lacunosum-moleculare. Those with right TLE exhibited similar ipsilateral hippocampal atrophy but with additional segmental volume depletion [33].

It is worth mentioning that there are not many similar types of software to volBrain. One of them is Neuroquant, which automatically measures the volume of brain structures. It has found application in observing changes in the course of Alzheimer’s disease, traumatic brain injury and multiple sclerosis [34]. There were also studies that analyzed the volume of brain structures in patients with epilepsy, but the study group consisted of adult patients [35]. However, one study compared neuroradiologist visual MR imaging analysis to Neuroquant analysis, and it turned out that they had similar specificity (90.4% versus 91.6%; *p* = 0.99), but Neuroquant had lower sensitivity (69.0% versus 93.0%, *p* < 0.001) [36]. Several studies have included children in research groups, but none have reported data relating to epilepsy patients. FreeSurfer is another available software that, among others, allows for volumetric segmentation of the majority of macroscopically visible brain structures [37]. Some evidence supports the possible application of this software in the case of epilepsy patients. Results of one of the studies indicated hippocampal reduction in patients with mesial temporal lobe epilepsy [38]. Similarly, in different studies, the volumes of hippocampal subfields were significantly lower on the ipsilateral side in a group of patients with temporal lobe epilepsy [39]. Our results were in line with both of the aforementioned studies.

Our study validates H1, H2 and H4 hypotheses. Healthy patients do not have any significant brain changes in comparison to children with epilepsy. The S group has lower values of cerebrum, cerebellum, brainstem, hippocampus, putamen, thalamus and nucleus accumbens, which indicates atrophy of the brain. Signs of atrophy were evident in the visual MR assessment (performed by an experienced radiologist) and then quantitatively confirmed by automated volumetric evaluation. Statistical analysis based on volBrain software is the basis for creating a parameterization. However, the H3 hypothesis is not fully validated due to the fact that there were no significant differences between the groups with and without the presence of comorbidities. Nonetheless, a declining trend was observed in the volumes of the majority of evaluated structures in patients with epilepsy in relation to healthy controls. In the case of lateral ventricles, the observed volumes were bigger in the epileptic subjects.

The data obtained have important clinical and prognostic significance; however, there are some limitations. First of all, they need to be confirmed in a large study group as this could significantly affect the results. In the study group, the presence of comorbidities was not significantly correlated, but there was a trend showing greater brain atrophy among patients with comorbidities. Other variables could be considered, such as disease duration and EEG abnormalities (the analysis has been conducted; however, no correlations were found with respect to the volume values of brain structures). Moreover, the interpretation of volumetry results is challenging due to the inherent methodological differences. Furthermore, normal morphometric differences are recognized in diverse populations that may need consideration. Our study did not assess the impact of the volume of individual parts of the CNS on cognitive functions. In addition, in the cited analyses, the research group consisted of only patients with specific types of epilepsy (drug-resistant epilepsy of the temporal lobe and the sclerosis of the hippocampal), while our study included all patients with symptoms of epilepsy regardless of the location.

## 5. Conclusions

Structural changes in people with epilepsy in the form of brain atrophy are well known; however, this is not assessed visually—there is a lack of parameterization. To our best knowledge, there are only a few studies that analyze the volumetry of particular brain structures, especially by using VolBrain software.

In our study, signs of atrophy were evident in the visual MR assessment (performed by an experienced radiologist) and then quantitatively confirmed by automated volumetric evaluation. Significant differences were evident, especially for structures such as brain tissue, lateral ventricles and putamen. Volumetric assessment of brain structures based on MR data can potentially be a useful diagnostic tool in children with epilepsy and can be implemented in clinical work for monitoring patients and the progression of the disease. In addition, it is a time-saving program and appears to be an effective, objective and accurate method; however, further studies are necessary to enhance the effectiveness of this software.

## Figures and Tables

**Table 1:** Difference between standard and epilepsy MR protocol.

Protocol	Sequence
Standard MR protocol	- T1-weighted sagittal, - T2-weighted axial, - fluid-attenuated inversion recovery (FLAIR) axial, - diffusion-weighted imaging or apparent diffusion coefficient
Epilepsy MR protocol	- examination of thin layers of the cerebral cortex in gradient echo - T2-weighted and FLAIR coronal/frontal oblique plane perpendicular to the long axis of hippocampus, - T1-weighted inversion recovery coronal oblique, - magnetization prepared rapid gradient echo, - susceptibility-weighted imaging, contrast-enhanced MR imaging [8,9]

**Table 2:** Normality test for the quantitative variables. Descriptive statistics (mean, median with lower and upper quartiles, min-max) with the statistical analysis (Shapiro–Wilk W test); statistically significant difference marked by “*” (asterisk).

Factor	Group	Mean (SD)	Me (Q1–Q3)	Min-Max	Test of Normality W (*p*)
WM	S	31.25% (3.68%)	32.00% (28.70–33.51%)	24.25–40.70%	0.98 (0.388)
GM	S	59.01% (3.87%)	58.89% (56.73–61.19%)	50.87–67.07%	0.98 (0.406)
Brain	S	90.25% (2.49%)	90.53% (88.25–91.60%)	86.14–96.96%	0.96 (0.069)
Cerebrum Total	S	78.89% (2.41%)	79.19% (77.02–80.45%)	74.99–85.05%	0.96 (0.067)
Cerebellum Total	S	9.91% (0.68%)	9.95% (9.45–10.28%)	8.31–11.97%	0.98 (0.647)
Brainstem Total	S	1.44% (0.18%)	1.44% (1.33–1.54%)	1.06–1.92%	0.99 (0.747)
Lateral ventricle Total	S	0.85% (0.49%)	0.73% (0.50–1.04%)	0.22–2.32%	0.89 (<0.001 *)
Caudate Total	S	0.56% (0.07%)	0.55% (0.51–0.60%)	0.43–0.79%	0.97 (0.132)
Putamen Total	S	0.63% (0.07%)	0.62% (0.58–0.67%)	0.47–0.79%	0.97 (0.127)
Thalamus Total	S	0.87% (0.06%)	0.88% (0.84–0.90%)	0.71–1.06%	0.95 (0.019 *)
Globus Pallidus Total	S	0.19% (0.03%)	0.19% (0.17–0.21%)	0.14–0.25%	0.96 (0.078)
Hippocampus Total	S	0.48% (0.06%)	0.49% (0.45–0.52%)	0.26–0.58%	0.88 (<0.001 *)
Amygdala Total	S	0.11% (0.02%)	0.11% (0.10–0.12%)	0.07–0.17%	0.94 (0.007 *)
Accumbens Total	S	0.05% (0.01%)	0.04% (0.04–0.05%)	0.02–0.07%	0.91 (<0.001 *)
WM	C	31.63% (4.65%)	32.98% (30.59–34.67%)	17.94–37.75%	0.87 (<0.001 *)
GM	C	59.50% (4.67%)	58.38% (56.11–62.46%)	52.04–68.45%	0.94 (0.070)
Brain	C	91.02% (2.54%)	91.89% (89.01–92.60%)	84.22–94.85%	0.92 (0.016 *)
Cerebrum Total	C	79.57% (2.63%)	80.32% (77.41–81.47%)	72.65–83.24%	0.93 (0.040 *)
Cerebellum Total	C	10.04% (0.82%)	10.07% (9.63–10.72%)	7.71–11.57%	0.97 (0.437)
Brainstem Total	C	1.49% (0.16%)	1.51% (1.34–1.61%)	1.08–1.72%	0.95 (0.161)
Lateral ventricle Total	C	0.55% (0.34%)	0.45% (0.28–0.70%)	0.20–1.44%	0.86 (<0.001 *)
Caudate Total	C	0.54% (0.06%)	0.54% (0.51–0.57%)	0.43–0.66%	0.97 (0.509)
Putamen Total	C	0.67% (0.07%)	0.65% (0.62–0.70%)	0.54–0.88%	0.94 (0.084)
Thalamus Total	C	0.90% (0.07%)	0.90% (0.86–0.95%)	0.78–1.05%	0.97 (0.399)
Globus Pallidus Total	C	0.19% (0.03%)	0.19% (0.17–0.22%)	0.13–0.25%	0.96 (0.208)
Hippocampus Total	C	0.51% (0.05%)	0.51% (0.48–0.53%)	0.39–0.62%	0.98 (0.629)
Amygdala Total	C	0.11% (0.01%)	0.11% (0.10–0.12%)	0.09–0.14%	0.93 (0.026 *)
Accumbens Total	C	0.05% (0.01%)	0.04% (0.04–0.05%)	0.03–0.08%	0.88 (0.001 *)

**Table 3:** Comparison of the volumes (% of IC) of particular brain structures between study and control groups. Descriptive statistics (N, median with lower and upper quartiles) with the statistical analysis (Mann–Whitney U test); statistically significant difference marked by “*” (asterisk).

Factor	Group (N) Me (Q1–Q3)		Statistical Analysis Z (*p*)
	Study (57)	Control (34)	
WM	32.00% (28.70–33.51%)	31.63% (30.59–34.67%)	−1.12 (0.263)
GM	58.89% (56.73–61.19%)	59.50% (56.11–62.46%)	−0.15 (0.879)
Brain	90.53% (88.25–91.60%)	91.02% (89.01–92.60%)	−2.19 (0.029 *)
Cerebrum Total	79.19% (77.02–80.45%)	79.57% (77.41–81.47%)	−1.78 (0.075)
Cerebellum Total	9.95% (9.45–10.28%)	10.04% (9.63–10.72%)	−1.04 (0.297)
Brainstem Total	1.44% (1.33–1.54%)	1.49% (1.34–1.61%)	−1.55 (0.121)
Lateral ventricle Total	0.73% (0.50–1.04%)	0.55% (0.28–0.70%)	3.25 (0.001 *)
Caudate Total	0.55% (0.51–0.60%)	0.54% (0.51–0.57%)	1.21 (0.226)
Putamen Total	0.62% (0.58–0.67%)	0.67% (0.62–0.70%)	−2.01 (0.044 *)
Thalamus Total	0.88% (0.84–0.90%)	0.90% (0.86–0.95%)	−1.73 (0.083)
Globus Pallidus Total	0.19% (0.17–0.21%)	0.19% (0.17–0.22%)	0 (0.997)
Hippocampus Total	0.49% (0.45–0.52%)	0.51% (0.48–0.53%)	−1.77 (0.076)
Amygdala Total	0.11% (0.10–0.12%)	0.11% (0.10–0.12%)	0.21 (0.834)
Accumbens Total	0.04% (0.04–0.05%)	0.05% (0.04–0.05%)	−0.2 (0.841)

**Table 4:** Comparing the values of particular variables in children with epilepsy. Descriptive statistics (N, median with lower and upper quartiles) with the statistical analysis (Mann–Whitney U test).

Factor	Me (Q1–Q3)		Statistical Analysis Z (*p*)
	Without Comorbidities (28)	With Comorbidities (18)	
White Matter	32.27% (30.29–34.03%)	30.38% (26.35–33.18%)	1.56 (0.118)
Grey Matter	58.03% (56.23–60.43%)	59.78% (56.94–64.46%)	−1.1 (0.270)
Brain	90.55% (88.33–91.62%)	90.11% (88.01–91.60%)	0.46 (0.645)
Lateral ventricle Total	0.69% (0.50–0.94%)	0.98% (0.59–1.16%)	−1.28 (0.200)
Hippocampus Total	0.50% (0.46–0.53%)	0.49% (0.47–0.52%)	−0.18 (0.857)

## Data Availability

The data that support the findings of this study are available on reasonable request from the corresponding author. The data are not publicly available due to privacy.
